# Albumin-fibrinogen ratio and fibrinogen-prealbumin ratio as promising prognostic markers for cancers: an updated meta-analysis

**DOI:** 10.1186/s12957-020-1786-2

**Published:** 2020-01-13

**Authors:** Da-wei Sun, Lin An, Guo-yue Lv

**Affiliations:** 1 Department of Hepatobiliary and Pancreatic Surgery, The First Hospital of Jilin University, Jilin University, Changchun, Jilin, 130021 China; 2 Department of Hand surgery, China-Japan Union Hospital of Jilin University, Jilin University, Changchun, Jilin, 130033 China

**Keywords:** Albumin-fibrinogen ratio (AFR), Fibrinogen-prealbumin ratio (FPR), Cancer, Prognostic, Meta-analysis

## Abstract

**Objective:**

Provide an updated and comprehensive evaluation of the prognostic value of the albumin-fibrinogen ratio (AFR) and the fibrinogen-prealbumin ratio (FPR) for patients with cancer.

**Materials and methods:**

Four databases (PubMed, Web of Science, Cochrane Library, and WanFang) were searched. The primary endpoints were overall survival (OS), disease-free survival (DFS), and progression-free survival (PFS). Pooled data were synthesized using StataMP 14 and expressed as hazard ratios (HRs) and 95% confidence intervals (CIs).

**Results:**

This update examined 19 studies (7282 cases) that assessed the correlation of AFR with cancer prognosis. Pooled univariate and multivariate analyses indicated significant correlations of low AFR with poor OS (HR 2.18, 95%CI 1.87–2.55 and HR 1.75, 95%CI 1.54–2.00, respectively), poor DFS (HR 1.89, 95%CI 1.54–2.32 and HR 1.51, 95%CI 1.29–1.76, respectively), and poor PFS (HR 1.68, 95%CI 1.42–1.99 and HR 1.48, 95%CI 1.16–1.88, respectively). Pooled univariate and multivariate analyses of 6 studies (2232 cases) indicated high FPR significantly correlated with poor OS (HR 2.37, 95%CI 2.03–2.77 and HR 1.97, 95%CI 1.41–2.77, respectively). One study reported that high FPR correlated with poor DFS (univariate analysis: HR 2.20, 95%CI 1.35–3.57; multivariate analysis: HR 1.77, 95%CI 1.04–2.99) and one study reported a correlation of high FPR with poor PFS in univariate analysis alone (HR 1.79, 95%CI 1.11–2.88).

**Conclusion:**

A low AFR and a high FPR correlated with increased risk of cancer mortality and recurrence. AFR and FPR may be promising prognostic markers for cancers.

## Introduction

Cancer incidence and mortality are rapidly increasing worldwide. There were an estimated 18.1 million newly diagnosed cancer cases and 9.6 million cancer deaths throughout the world during 2018 [1]. Research indicates that during the twenty-first century cancer will be the second-leading cause of death in the USA [2] and the single most important barrier to increasing life expectancy worldwide [1]. Moreover, the 5-year relative survival rate for all cancers is only 67%, thus indicating that prognoses of patients with cancer remain poor [3]. Therefore, evidenced-based resources are needed to improve survival from cancer and to identify patient characteristics that affect prognosis.

Malnutrition is common in cancer patients and is associated with increased morbidity and mortality [4]. Serum levels of albumin and prealbumin are common indicators of nutritional status. Albumin, which normally accounts for more than 50% of blood protein, is synthesized and secreted from the liver, and its concentration reflects the protein status of the blood and internal organs [5]. It functions as the major modulator of plasma oncotic pressure, and it also transports a variety of substances, including endogenous physiological metabolites and exogenous ligands [6]. The pretreatment serum albumin level in a cancer patient is generally used to assess nutritional status and predict prognosis [7].

Prealbumin, also known as transthyretin, has a much shorter half-life and smaller serum pool than albumin. Its main functions are to bind and transport endogenous proteins and small molecules. Prealbumin is more sensitive to changes in protein-energy status than albumin, and its concentration closely reflects recent dietary intake rather than overall nutritional status [8]. Prealbumin concentration can therefore be regarded primarily as a marker of at-risk patients who require nutritional monitoring [9]. In particular, the prealbumin level provides a quantitative measure of the efficacy of a nutritional care plan and an indication of the need to modify interventions [5].

Clinicians have long recognized specific associations of hemostatic system disorders with diverse cancers. The polypeptide fibrinogen is the central protein in the hemostasis pathway and occurs as a deposit in most tumors that occur in humans and experimental animals [10]. Fibrinogen is a 340 kDa hexameric plasma glycoprotein synthesized by the liver and consists of three pairs of non-identical polypeptide chains, the α-, β-, and γ-chains [11]. Fibrinogen deposit within the tumor stroma, especially the extracellular matrix, serves as a scaffold that supports the binding of growth factors which promote cellular responses associated with tumor cell adhesion, proliferation, and migration during cell growth and angiogenesis [12]. An elevated serum fibrinogen level is commonly associated with poor overall survival (OS) in human cancers [13].

Previous meta-analyses of cancer patients indicated that several ratios of pretreatment systemic inflammatory markers or/and nutritional markers, such as the neutrophil-lymphocyte ratio (NLR) [14], the albumin-globulin ratio (AGR) [15], and the C-reactive protein-albumin ratio (CAR) [16], can predict prognosis. Recent studies have examined the albumin-fibrinogen ratio (AFR) and/or the fibrinogen-prealbumin ratio (FPR) as prognostic indicators in cancer. For instance, a previous meta-analysis of 12 studies found that a low AFR was associated with poor prognosis in cancer [17]. However, this meta-analysis only assessed the impact of AFR on OS via multivariate analytic results and only evaluated the impact of AFR on disease-free survival (DFS) based on two studies. The impact of AFR on progression-free survival (PFS) and the impact of FPR on prognosis of cancer patients remain unknown. We conducted the present updated meta-analysis to elucidate the role of AFR in cancer prognosis and included all newly available studies to identify the prognostic value of FPR in cancers.

## Materials and methods

### Search strategy

Potential studies were identified by searching four online databases (PubMed, Web of Science, Cochrane Library, and WanFang) using PRISMA guidelines [18]. All studies that were published up to Oct 22, 2019, were identified using the search terms: “albumin fibrinogen ratio” or “fibrinogen albumin ratio” or “fibrinogen prealbumin ratio” or “prealbumin fibrinogen ratio.” The reference lists of initially identified studies were also scrutinized to identify additional relevant studies.

### Criteria for eligibility

Eligibility criteria were defined according to the Population, Intervention, Comparison, Outcome and Study Design (PICOS) strategy. Thus, “Population” refers to cases diagnosed with cancers; “Intervention” refers to low AFR or high FPR; “Comparison” refers to the control group (without low AFR or high FPR); “Outcome” refers to OS, DFS, or PFS; and “Study design” refers to a prospective or retrospective study. A study was deemed ineligible if it was a duplicate, a review article, a comment or letter, or a case series. When there were duplicate records, the most recently published record was used.

### Data collection

Data were collected independently by two authors using a predefined form. These forms were further checked by the third author, and disagreement was reconciled by consensus among all authors. The data extracted from each article included author names, publication year, country, cancer category, cancer stage, study design (prospective or retrospective), primary treatment option, optimal cut-off value and method used to select the optimal cut-off value, number of cases (total and numbers above and below the cut-off value), prognostic outcome, hazard ratios (HRs) with 95% confidence intervals (CIs), method of data analysis, data source (crude data or fitted curve), and follow-up interval. HR data were extracted from univariate and multivariate analyses if available.

### Statistical analysis

When prognostic outcomes were provided as Kaplan-Meier curves, Engauge Digitizer 4.1 software was used to read the curves and identify the times of deaths. These data, defined as time-event outcomes, were used to calculate HRs and 95% CIs using the method of Tierney et al. [19]. These data were then synthesized and expressed as HRs with 95% CIs using StataMP 14. Cochran’s *Q* and *I*^2^ statistics were utilized to assess the heterogeneity among included studies [20]. When there was significant heterogeneity (*P* < 0.1 and/or *I*^2^ > 50%), pooled data were analyzed using a random-effects model; otherwise, a fixed-effects model was used. All statistical tests were two-sided, and a *P* value less than 0.05 was deemed statistically significant. The statistical analyses, stratification analyses, and sensitivity analyses were the same as those used in our previous publications [15, 21].

## Results

### Study selection

**Fig. 1:**
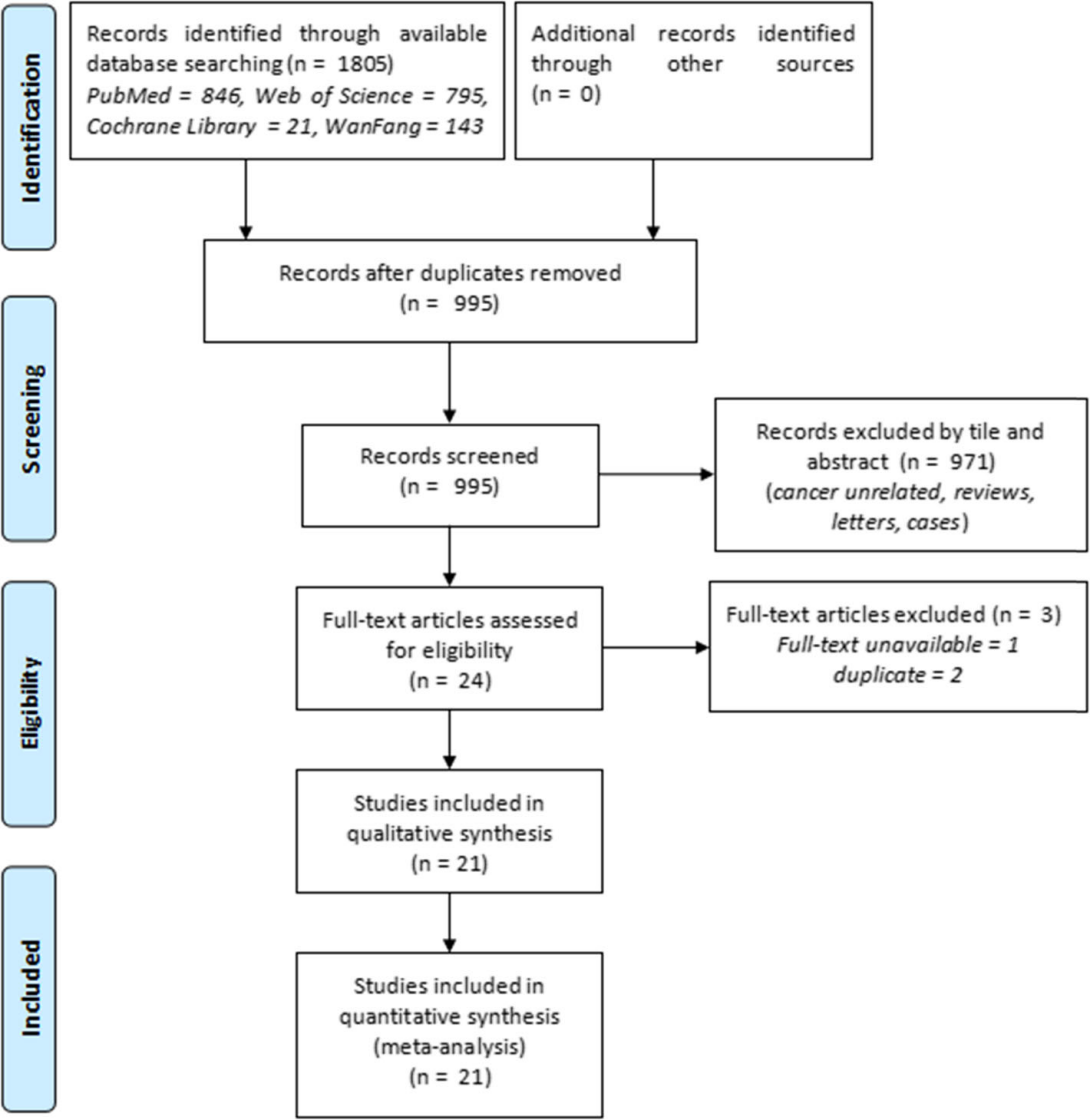
Procedure used to identify studies for inclusion in the meta-analysis (PRISMA guidelines)

We initially identified 1805 records, and 995 of these records remained after removal of duplicates (Fig. 1). After review of the titles and abstracts, we excluded an additional 971 records. We reviewed the full text of 24 records and ultimately included 21 studies in the quantitative analysis [22–42].

### Characteristics of included studies

Nineteen of the included studies evaluated the role of AFR in cancer prognosis, 7 more studies than examined in the previous meta-analysis of the prognostic value of AFR in cancer [17]. In addition, 7 of the included studies examined the prognostic value of FPR in cancer prognosis. We thoroughly evaluated the prognostic value of the AFR and FPR in cancers by analysis of OS, DFS, and PFS (Table 1).

**Table 1:** Characteristics of studies included in the meta-analysis

Author [Ref.]	Year	Country	Cancer category	Case no.	Cancer stage	Design type	Primary treatment option	Optimal cut-off for AFR/FPR	No. > against No. < (cut-off)	Prognostic outcomes	Hazard ratio (95% CI)	Data origin	Follow-up period (months)
Chen et al. [22]	2019	China	Colorectal cancer	430	Metastatic	Prospective	Mixed modality but targeted therapy	9.9 by X-tile (AFR)	NR	OS, PFS	OS(U),1.73 (1.35–2.21), Cohort 1	Crude	More than 36
											PFS(U),1.85 (1.29–2.65), Cohort 1	Crude	
Chen et al. [22]	2019	China	Colorectal cancer	77	Metastatic	Prospective	Radiochemotherapy	9.9 by X-tile (AFR)	NR	OS, PFS	OS(U), 1.75 (0.93–3.31), Cohort 2	Crude	More than 36
											PFS(U),1.43 (0.84–2.42), Cohort 2	Crude	
Yu et al. [23]	2019	China	Ovarian cancer	313	Advanced	Retrospective	Surgery plus chemotherapy	7.78 by ROC (AFR)	162/151	OS, PFS	OS(U), 2.50 (1.44–4.09)	Crude	At least 12
											OS(M),2.19 (1.18–3.67)	Crude	
											PFS(U),1.56 (1.21–2.05)	Crude	
											PFS(M),1.38 (1.04–1.82)	Crude	
Zhang et al. [24]	2019	China	Colorectal cancer	71	Metastatic	Retrospective	Chemotherapy	10.63 by ROC (AFR)	23/48	PFS	PFS(U),1.91 (1.14–3.20)	Crude	Median 6.67 (1.86-27.17)
Li et al. [25]	2019	China	Ovarian cancer	186	I–IV (FIGO)	Retrospective	Surgical resection	Score = 0 (AFR)	148/38	OS	OS(U),1.92 (1.56–2.23), Subgroup 1	Crude	Median 45.5 (2.0-45.5)
											*OS(M),1.05 (1.02*–*1.63), Subgroup 1*	*Crude*	
											OS(U),2.53 (1.36–4.71), Subgroup 2	Crude	
											OS(M),2.13 (1.14–3.26),Subgroup 2	Crude	
Ying et al. [26]	2019	China	NSCLC	270	III–IV (TNM)	Retrospective	Chemotherapy	8.02 by ROC (AFR)	119/151	OS, PFS	OS(U),1.93 (1.28–2.98)	Crude	Up to 60
											OS(M),1.79 (1.11–2.59)	Crude	
											PFS (U), 2.07 (1.32–3.12)	Crude	
											PFS (M),1.80 (1.09-2.78)	Crude	
Du [27]	2019	China	Gallbladder cancer	220	Metastatic	Retrospective	Chemotherapy mainly	15.45 by X-tile (AFR)	NR	OS	OS(U),1.97 (0.96–3.67)	Crude	More than 36
											OS(M),0.89 (0.46–1.73)	Crude	
Wang [28]	2019	China	CRLM	452	Metastatic	Retrospective	Surgical resection	13.16 by X-tile (AFR)	260/192	OS, DFS	OS(U),1.71 (1.30–2.25)	Crude	Median 28
											OS(M),1.63 (1.16–2.30)	Crude	
											DFS(U),1.60 (1.28–2.00)	Crude	
											DFS(M),1.45 (1.14–1.88)	Crude	
Chen et al. [29]	2018	China	NSCLC	529	I–III (AJCC)	Retrospective	Surgical resection	9.67 by ROC (AFR)	392/137	OS, DFS	OS(U),4.22 (2.83–6.29)	Crude	Median 35.0 (1–78.5)
											OS(M),1.95 (1.21–3.16)	Crude	
											DFS(U),2.73 (2.06–3.62)	Crude	
											DFS(M),1.78 (1.27–2.51)	Crude	
Gao et al. [30]	2018	China	ESCC	153	0–III (AJCC)	Prospective	Surgical resection	9.3 by ROC (AFR)	128/25	OS	OS(U),1.95 (1.18–3.25)	Crude	More than 36
											OS(M),2.38 (1.15–4.93)	Crude	
Li et al. [31]	2018	China	Lung cancer	412	I–IV	Prospective	Multiple modality	7.8 by ROC (AFR)	NR	OS	OS(U),1.97 (1.36–2.85)	Crude	More than 36
											OS(M),1.79 (1.23–2.61)	Crude	
Sun et al. [32]	2018	China	Colorectal cancer	702	I–III (AJCC)	Prospective	Surgical resection	9.2 by X-tile (AFR)	562/118	OS	OS(U),1.88 (1.25–2.84)	Crude	More than 36
											OS(M),1.41 (0.90–2.20)	Crude	
Liang et al. [33]	2018	China	Soft tissue sarcoma	310	IA–IV (AJCC)	Retrospective	Surgical resection	13.77 by ROC (AFR)	176/134	OS, DFS	OS(U),3.15 (2.07–4.79)	Crude	Median 91.5
											OS(M),1.91 (1.16–3.13)	Crude	
											DFS(U),1.96 (1.41–2.72)	Crude	
Xu et al. [34]	2018	China	HCC	151	0-C (BCLC)	Retrospective	Surgical resection	16.1 by ROC (AFR)	50/101	OS, DFS	OS(U),2.15 (1.35–3.40)	Crude	Median 33.8 (1–86)
											OS(M),2.02 (1.27–3.21)	Crude	
											DFS(U),1.70 (1.14–2.54)	Crude	
											DFS(M),1.56 (1.03–2.35)	Crude	
Sun et al. [35]	2018	China	ESCC	373	I–III (AJCC)	Retrospective	Surgical resection	Score = 0 (AFR)	154/219	OS, DFS	OS(U),1.69 (1.27–2.24)	Crude	Median 51.9
											OS(M),1.37 (1.01–1.88)	Crude	
											DFS(U),1.68 (1.28–2.20)	Crude	
											DFS(M),1.39 (1.04–1.88)	Crude	
Xu et al. [36]	2018	China	Gallbladder cancer	154	0–IVB (AJCC)	Retrospective	Surgical resection	12.5 by ROC (AFR)	71/83	OS	OS(U),4.63 (2.99–7.17)	Crude	Median 17
											OS(M),2.81 (1.77–4.48)	Crude	
Zou et al. [37]	2018	China	Leukemia	191	A–C (Binet stage)	Retrospective	Untreated	9.7 by X-tile (AFR)	171/20	OS	OS(U),3.65 (1.67–7.99)	Crude	Median 51 (1–270)
											OS(M),3.47 (1.54–7.82)	Crude	
											PFS (U),1.24 (0.72–2.13)	Crude	
Zhang et al. [38]	2017	China	Gastric cancer	360	II–III (AJCC)	Retrospective	Surgical resection	8.9 by X-tile (AFR)	290/70	OS	OS(U),2.34 (1.59–3.45)	Crude	More than 36
											OS(M),1.54 (1.01–2.34)	Crude	
Hwang et al. [39]	2017	Korea	Breast cancer	793	I–III (AJCC)	Retrospective	Surgical resection	14.08 by ROC (AFR)	538/255	OS	OS(U),2.72 (1.66–4.47)	Crude	Median 44.0 (0–197)
											OS(M),2.62 (1.46–4.72)	Crude	
Tan et al. [40]	2017	China	ESCC	1135	T1-4aN0-3 (AJCC)	Retrospective	Surgical resection	12.5 by X-tile (AFR)	625/510	OS	OS(U),1.38 (1.22–1.56)	Curve	More than 60
											OS(M),1.33 (1.09–1.69)	Crude	
Zhang et al. [41]	2019	China	HCC	230	A–C (BCLC)	Prospective	Surgical resection	*15.6 by X-tile (FPR)*	NR	OS, DFS	OS(U),5.07 (2.55–10.06)	Crude	More than 36
											OS(M),4.16 (2.06–8.39)	Crude	
											DFS(U),2.20 (1.36–3.57)	Crude	
											DFS(M),1.77 (1.04–2.99)	Crude	
Li [42]	2019	China	NSCLC	360	IIB–IV (AJCC)	Retrospective	Chemotherapy	*21.24 by ROC (FPR)*	151/209	OS	OS(U),2.09 (1.66–2.62)	Data	3–45
											OS(M),1.22 (1.04–1.43)	Crude	
Du et al. [27]	2019	China	Gallbladder cancer	220	Metastatic	Retrospective	Chemotherapy mainly	*31.84 by X-tile (FPR)*	NR	OS	OS(U),1.93 (1.26–2.97)	Crude	More than 36
											OS(M),1.57 (1.00–2.46)	Crude	
Sun et al. [32]	2018	China	Colorectal cancer	555	I–III (AJCC)	Prospective	Surgical resection	*18.3 by X-tile (FPR)*	230/325	OS	OS(U),2.40 (1.57–3.67)	Crude	More than 36
											OS(M),1.94 (1.24–3.05)	Crude	
Zhang et al. [38]	2017	China	Gastric cancer	360	II–III (AJCC)	Retrospective	Surgical resection	*12.1 by X-tile (FPR)*	246/114	OS	OS(U),3.37 (2.02–5.64)	Crude	More than 36
											OS(M),2.33 (1.37–3.94)	Crude	
Chen et al. [22]	2019	China	Colorectal cancer	430	Metastatic	Prospective	Mixed modality but targeted therapy	*22.8 by X-tile (FPR)*	NR	OS	OS(U),2.33 (1.42–3.82), Cohort 1	Crude	More than 36
											OS(M),1.90 (1.10–3.28), Cohort 1	Crude	
Chen et al. [22]	2019	China	Colorectal cancer	77	Metastatic	Prospective	Radiochemotherapy	*22.8 by X-tile (FPR)*	NR	OS	OS(U),4.47 (1.65–12.14), Cohort 2	Crude	More than 36
											OS(M),4.21 (1.16–15.27), Cohort 2	Crude	
Zhang et al [24]	2019	China	Colorectal cancer	71	Metastatic	Retrospective	Chemotherapy	*18.49 by ROC (FPR)*	23/48	PFS	PFS(U),1.79 (1.11–2.88)	Crude	Median 6.67 (1.86–27.17)

*NSCLC* non-small cell lung cancer, *CRLM* colorectal liver metastases, *ESCC* esophageal squamous cell carcinoma, *HCC* hepatocellular carcinoma, *AFR* albumin to fibrinogen, *FPR* fibrinogen to prealbumin ratio, *NR* not reported, *ROC* receiver operating characteristic, *U* univariate, *M* multivariate

### Effect of AFR on OS, DFS, and PFS

**Fig. 2:**
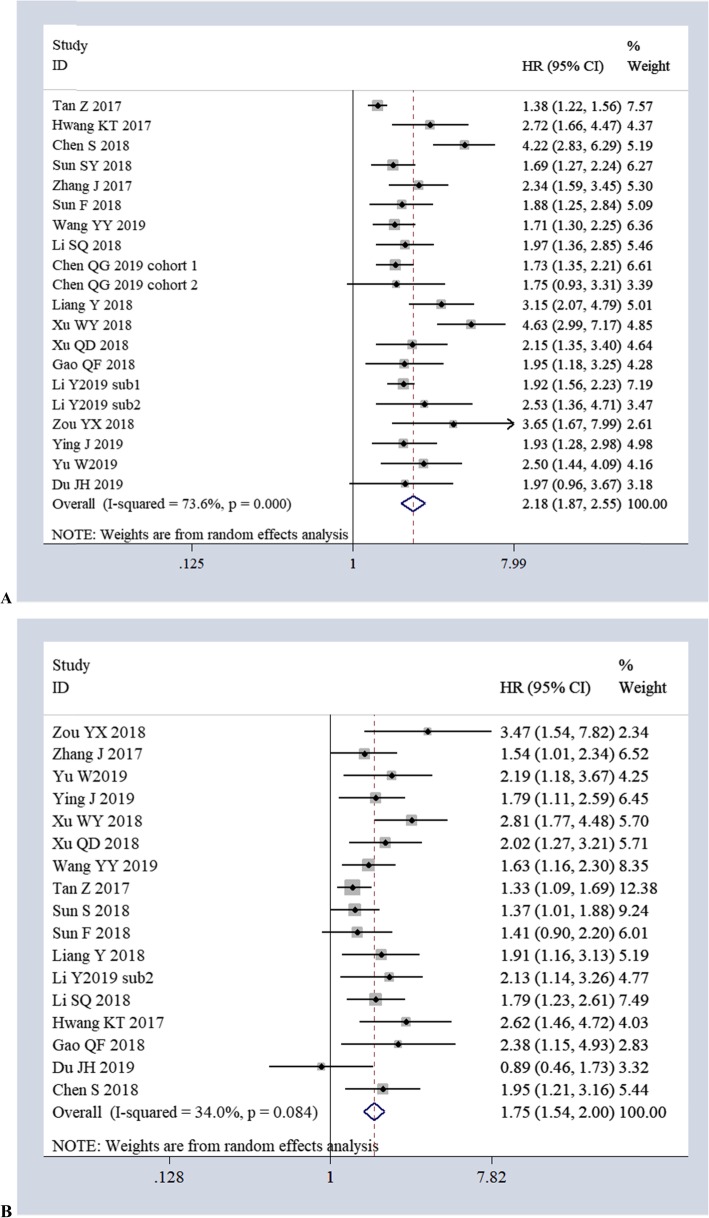
Forest plots of the relationship between AFR and OS *via* univariate analyses (**a**) and multivariate analyses (**b**)

The association of AFR with OS was reported in 18 studies (7211 cases) using univariate analysis and in 17 studies (6704 cases) using multivariate analysis. Based on the univariate analyses, the pooled results of a random-effects model (*I*^2^ *= 73.6%, P = 0.000*) showed a significant association between low AFR and poor OS (*HR 2.18, 95% CI 1.87–2.55, P = 0.00*) (Fig. 2a). Based on the multivariate analyses, the pooled results of a random-effects model (*I*^2^ *= 34.0%, P = 0.084*) also showed a significant association between low AFR and poor OS (*HR 1.75, 95% CI 1.54–2.00, P = 0.00*) (Fig. 2b).

**Fig. 3:**
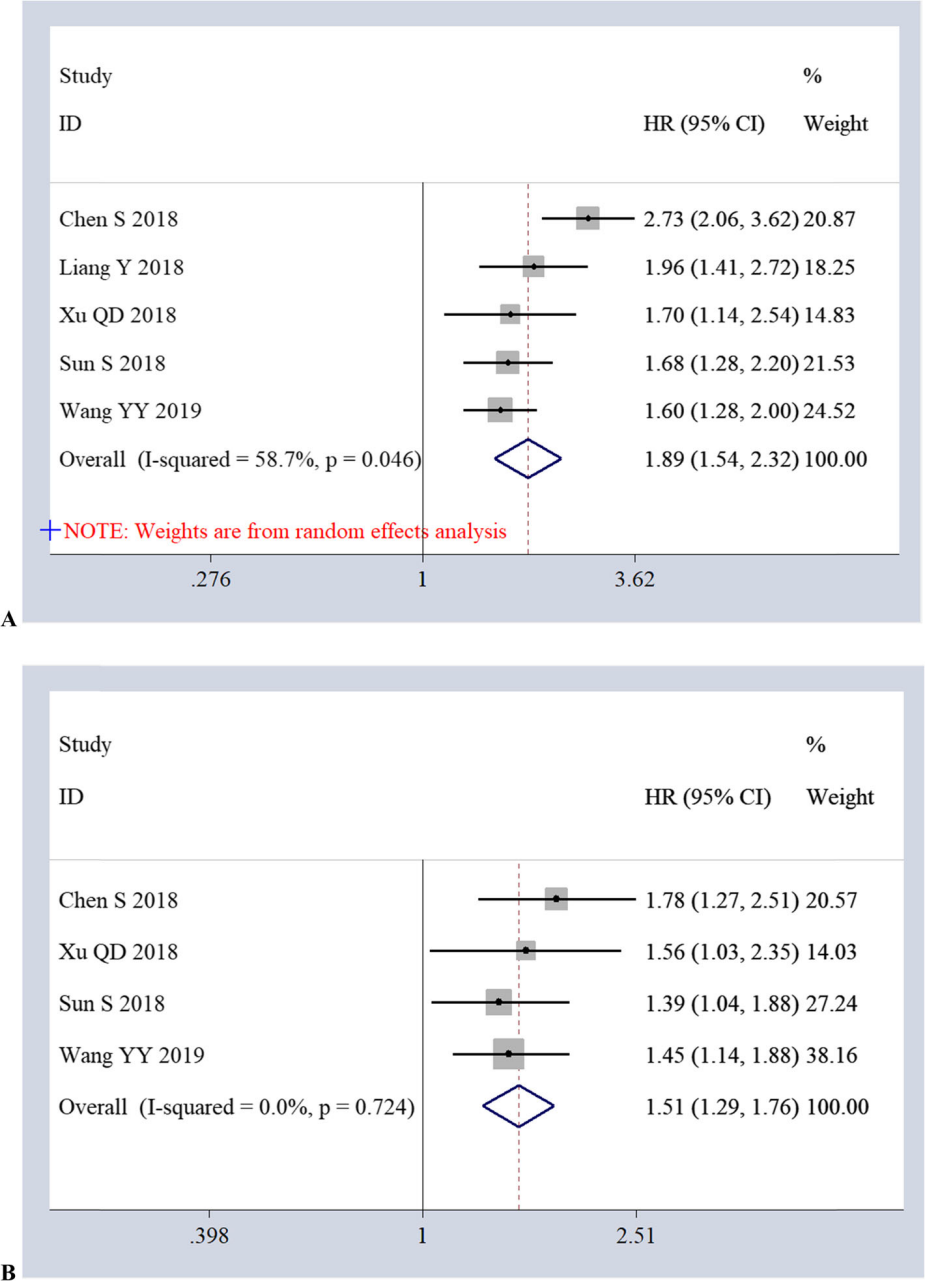
Forest plots of the relationship between AFR and DFS *via* univariate analyses (**a**) and multivariate analyses (**b**)

The association of AFR with DFS was reported in 5 studies (1815 cases) using univariate analysis and in 4 studies (1505 cases) using multivariate analysis. Based on the univariate analyses, the pooled results of a random-effects model (*I*^2^ *= 58.7%, P = 0.046*) demonstrated a significant association between low AFR and poor DFS (*HR 1.89, 95%CI 1.54–2.32, P = 0.00*) (Fig. 3a). Based on the multivariate analyses, the pooled results of a fixed-effects model (*I*^2^ *= 0.0%, P = 0.724*) also showed a correlation of low AFR with poor DFS (*HR 1.51, 95%CI 1.29–1.76, P = 0.00*) (Fig. 3b).

**Fig. 4:**
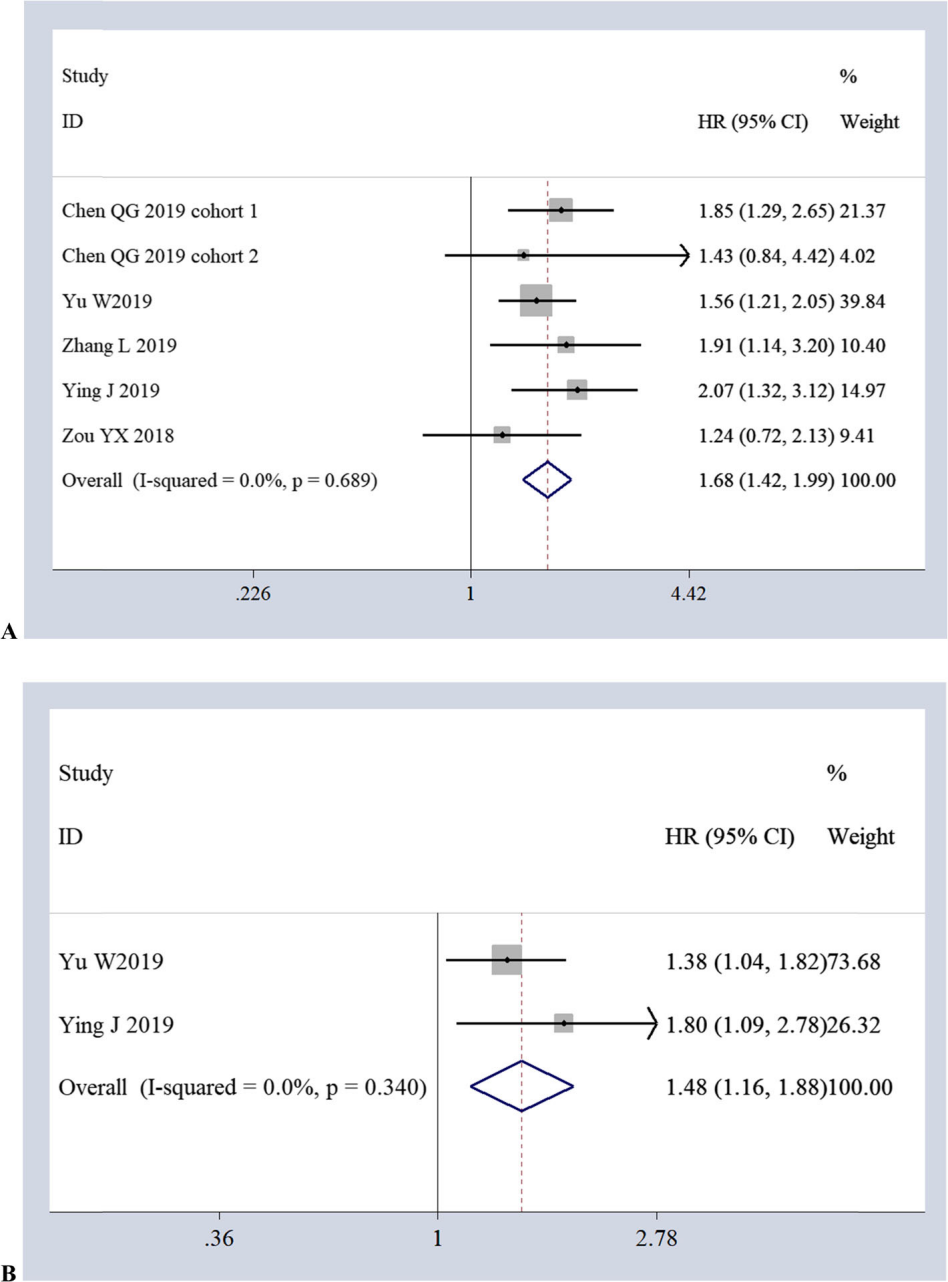
Forest plots of the relationship between AFR and PFS *via* univariate analyses (**a**) and multivariate analyses (**b**)

The association of AFR with PFS was reported in 6 studies (1352 cases) using univariate analysis and in 2 studies (583 cases) using multivariate analysis. According to a fixed-effects model, meta-analysis showed that low AFR was associated with poor PFS in the univariate analyses (*HR 1.68, 95%CI 1.42–1.99, P = 0.00; I*^2^ *= 0.0%, P = 0.689*) (Fig. 4a) and in the multivariate analyses (*HR 1.48, 95%CI 1.16–1.88, P = 0.00; I*^2^ *= 0.0%, P = 0.340*) (Fig. 4b).

### Effect of FPR on OS, DFS, and PFS

**Fig. 5:**
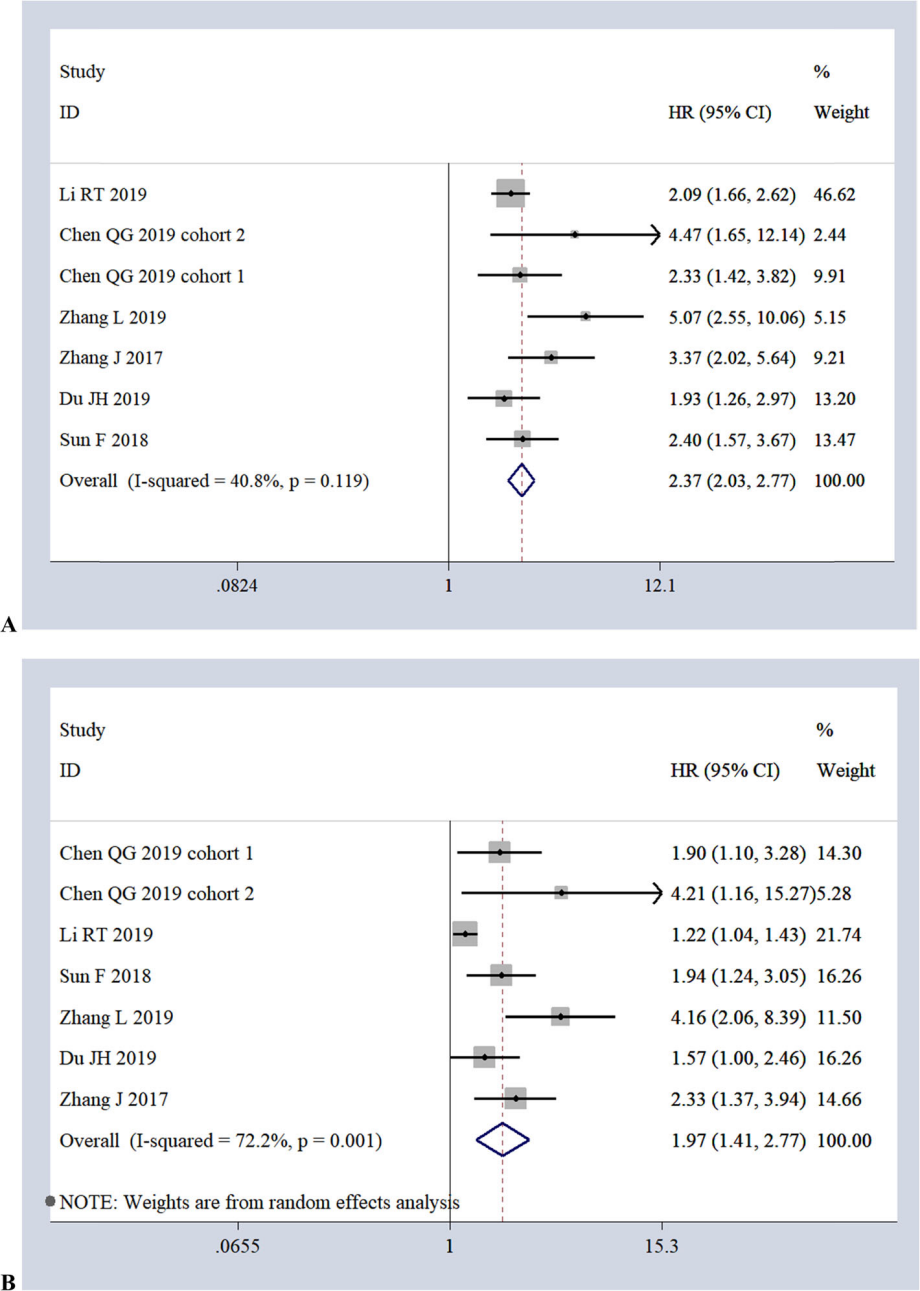
Forest plots of the relationship of FPR with OS *via* univariate analyses (**a**) and multivariate analyses (**b**)

The correlation of FPR with OS was evaluated in 6 studies (2232 cases) using both univariate and multivariate analyses. The pooled data of a fixed-effects model (*I*^2^ *= 40.8%, P = 0.119*) indicated a significant association between high FPR and poor OS in the univariate analysis (*HR 2.37, 95%CI 2.03–2.77, P = 0.00*) (Fig. 5a). The pooled data of a random-effects model (*I*^2^ *= 72.2%, P = 0.001*) also showed a significant relationship between high FPR and poor OS in the multivariate analysis (*HR 1.97, 95%CI 1.41–2.77, P = 0.00*) (Fig. 5b). Only two studies evaluated the correlation of FPR with DFS [41] and PFS [24], so we did not perform a pooled meta-analysis of these results. One of these studies examined 230 cases of hepatocellular carcinoma (HCC) and found a significant association between high FPR and poor DFS based on univariate analysis (*HR 2.20, 95%CI 1.35–3.57, P = 0.001*) and multivariate analysis (*HR 1.77, 95%CI 1.04–2.99, P = 0.034*). The other study examined 71 cases of metastatic colorectal cancer and found a significant association between high FPR and poor PFS (*HR 1.79, 95%CI 1.11–2.88, P = 0.017*) based on univariate analysis alone.

### Subgroup meta-analysis for AFR and OS

In this update, there was heterogeneity among the studies that examined the relationship of AFR with OS. Thus, we performed subgroup analyses based on the AFR cut-off value, methods of choosing the cut-off value, study design, number of cases, cancer classification, publication time, treatment option, and data source. Our results indicated that the relationship between AFR and OS remained despite variation of these factors. At the same time, the heterogeneity was eliminated in some of the subgroup meta-analyses when classified by these factors (Tables 2 and 3).

**Table 2:** Subgroup meta-analyses of the relationship between AGR and OS via univariate analyses

Potential confounding factor	No. of studies	No. cases	Hazard ratio with 95%CI	*P* value	*I*^2^ (%) for heterogeneity	*P* value for heterogeneity
Overall survival (OS)	18	7211	2.18 (1.87–2.55)	0.000	73.6	0.000
Methods for choosing AFR cut-off value						
X-tile	7	3567	1.78 (1.49–2.13)	0.000	53.4	0.036
Score	2	559	2.67 (2.13–3.36)	0.000	57.8	0.015
ROC	9	3085	1.88 (1.63–2.18)	0.000	0.0	0.479
Cut-off value of AFR						
> 9.7	8	3722	2.15 (1.65–2.80)	0.000	81.9	0.000
≤ 9.7	8	2930	2.38 (1.91–2.95)	0.000	45.7	0.075
Score = 0	2	559	1.88 (1.63–2.18)	0.000	0.0	0.479
Study designed type						
Retrospective	14	5437	2.33 (1.91–2.85)	0.000	80.4	0.000
Prospective	4	1774	1.83 (1.55–2.16)	0.000	0.0	0.978
Number of cases						
< 360	9	1948	2.45 (2.00–3.02)	0.000	51.9	0.028
≥ 360	9	5263	1.97 (1.61–2.41)	0.000	76.2	0.000
Cancer classification						
Lung cancers	3	1211	2.52 (1.52–4.17)	0.000	79.2	0.008
Digestive cancers	10	4207	1.94 (1.60–2.35)	0.000	71.6	0.000
Gynecological cancers	2	499	2.01 (1.71–2.36)	0.000	0.0	0.484
Other cancers	3	1294	3.05 (2.27–4.11)	0.000	0.0	0.806
Publication time						
After 2019	6	1948	1.88 (1.68–2.10)	0.000	0.0	0.880
Before 2019	12	5263	2.40 (1.85–3.11)	0.000	84.0	0.000
Treatment option						
Surgical resection	12	5298	2.26 (1.84–2.79)	0.000	82.1	0.000
Others	6	1913	1.94 (1.65–2.27)	0.000	0.0	0.629
HR source						
Crude data	17	6076	2.24 (1.96–2.57)	0.000	55.7	0.002
Curve estimation	1	1135	1.38 (1.22–1.56)	0.000	–	–

**Table 3:** Subgroup meta-analyses of the relationship between AGR and OS via multivariate analyses

Potential confounding factor	No. of studies	No. cases	Hazard ratio with 95%CI	*P* value	I^2^ (%) for Heterogeneity	*P* value for Heterogeneity
Overall survival (OS)	17	6704	1.75 (1.54–2.00)	0.000	34.0	0.084
Methods for choosing AFR cut-off value						
X-tile	6	3060	1.47 (1.19–1.81)	0.000	34.6	0.177
ROC	9	3085	2.07 (1.76–2.43)	0.000	0.0	0.886
Score	2	559	1.62 (1.06–2.47)	0.024	50.2	0.156
Cut-off value of AFR						
> 9.7	7	3215	1.76 (1.36–2.28)	0.000	61.4	0.017
≤ 9.7	8	2930	1.82 (1.53–2.15)	0.000	0.0	0.623
Score = 0	2	559	1.62 (1.06–2.47)	0.024	50.2	0.156
Study designed type						
Retrospective	14	5437	1.78 (1.52–2.08)	0.000	42.6	0.046
Prospective	3	1267	1.71 (1.31–2.23)	0.000	0.0	0.457
Number of cases						
< 360	9	1948	2.04 (1.66–2.50)	0.000	21.5	0.252
≥ 360	8	4756	1.53 (1.35–1.74)	0.000	2.7	0.409
Cancer classification						
Lung cancers	3	1211	1.83 (1.44–2.33)	0.000	0.0	0.956
Digestive cancers	10	3700	1.58 (1.32–1.90)	0.000	43.9	0.075
Gynecological cancers	2	499	2.16 (1.47–3.17)	0.000	0.0	0.944
Other cancers	3	1294	2.37 (1.68–3.34)	0.000	0.0	0.431
Publication time						
After 2019	5	1441	1.70 (1.33–2.17)	0.000	23.9	0.262
Before 2019	12	5263	1.79 (1.52–2.11)	0.000	42.0	0.062
Treatment option						
Surgical resection	12	5298	1.73 (1.50–2.01)	0.000	33.4	0.123
Others	5	1406	1.80 (1.31–2.49)	0.000	45.3	0.120

### Sensitivity analysis

In the initial meta-analysis of the relationship of AFR and OS from the multivariate analyses (Additional file 1), an apparently paradoxical plot (using crude HR with 95% CI in the original study) was present in one subgroup of the study by Li et al. [25]. Therefore, we deleted this subgroup during the meta-analysis. The sensitivity analysis (Additional files 2 and 3) indicated that all the included studies were nearly close to the central line, except the study by Li et al. [42]. In addition, the results of the sensitivity analysis indicated that omitting any single study did not change the overall effects of each pooled meta-analysis.

## Discussion

Cancer is a devastating disease, and patients typically have poor prognoses. Therefore, research is needed to identify novel prognostic factors, because these factors may help to improve risk stratification and lifestyle decisions of these patients [43].

We assessed the value of ratio indexes derived from serum albumin, prealbumin, and fibrinogen—AFR and FPR—as prognostic markers for human cancers in this updated meta-analysis. Relative to the previous meta-analysis [17], this update has two strengths. First, we included 7 more studies that examined the relationship between AFR and cancer prognosis, and we also evaluated the impact of AFR on OS, DFS, and PFS using the pooled results from univariate and multivariate analyses. Second, we identified 7 additional studies that evaluated FPR as a prognostic marker in human cancers. The pooled results indicated that a high FPR correlated with poor OS, poor DFS, and poor PFS. These results thus indicated that a low AFR and a high FPR correlated with an increased risk of cancer mortality and recurrence.

We must note that the values of the AFR and FPR indexes themselves do not affect the survival outcomes of cancer patients. Instead, the underlying proteins (albumin, prealbumin, and fibrinogen) and biological processes that determine the AFR and FPR are responsible for this relationship. Serum albumin and prealbumin are two of the most commonly used indicators for assessing malnutrition, and malnutrition adversely affects the outcomes of cancer patients, in that it increases the incidence of infections, the length of hospital stay, and the risk of death [44]. However, serum albumin level is also reduced in patients with locally advanced or metastatic malignancies irrespective of the presence of malnutrition [45]. In these patients, a low albumin level has an adverse influence on the outcome of anticancer therapy [46]. Inflammation also affects the visceral synthesis of albumin and prealbumin. As a key regulator of inflammation [47], fibrinogen can induce tumor angiogenesis and metastasis by directly interacting with endothelial cells, by indirectly interacting with other regulators of angiogenesis [10], and by enhancing tumor cell invasion and metastasis through epithelial-to-mesenchymal transition (EMT) signaling [48].

Although albumin/prealbumin, and fibrinogen abnormalities are well-documented prognostic markers in cancer patients, not all cancer patients suffer from deficiencies of albumin/prealbumin and an overabundance of fibrinogen; some patients only have an albumin/prealbumin deficiency or only a fibrinogen overabundance. The ratio indexes that we used—AFR and FPR—better reflect the levels of both albumin/prealbumin and fibrinogen. That is to say, the AFR and FPR are better markers of alterations of albumin/prealbumin (representing nutrition) and fibrinogen (representing hemostasis or inflammation).

Additionally, serum albumin, prealbumin, and fibrinogen are available in the medical records of most cancer patients, and measurements are inexpensive and reproducible. Thus, use of the AFR and FPR as prognostic markers in cancers has great potential. In summary, both AFR and FPR could be promising markers of cancer prognosis. These results may help to guide future cancer treatments by identifying sub-populations with different prognoses.

There were some weaknesses in this updated meta-analysis. The main weakness is that the relationship of FPR on DFS and PFS was based on only one included study, rather than a meta-analysis. Second, there was heterogeneity among the studies included, and our pooled results were nearly all based on random-effects models. Differences in the baseline values and characteristics of patients, treatment options, and cut-off values, and other factors among studies may account for this heterogeneity. Third, there was publication bias regarding the relationship between AFR with OS (more than 10 studies), though we did not present these results or funnel plots.

## Conclusions

A low AFR and a high FPR correlated with an increased risk of cancer mortality and recurrence. Thus, AFR and FPR may be promising prognostic markers for cancers.

## Supplementary information

**Additional file 1. **Forest plots of the relationship between AFR and OS *via* multivariate analyses, before deleting the subgroup with paradoxical results (Li et al. 2019, subgroup 1).

**Additional file 2. **Sensitivity analysis of the correlation of AFR with OS *via* univariate analyses (A) and multivariate analyses (B); with DFS from univariate analyses (C) and multivariate analyses (D); and with PFS from univariate analyses results (E) and multivariate analyses (F).

**Additional file 3. **Sensitivity analysis of the correlation of FPR with OS *via* univariate analyses (A) and multivariate analyses (B).

## Data Availability

The data used and analyzed in the current study are available from the corresponding author upon reasonable request.

## Abbreviations

AFR: Albumin to fibrinogen
CRLM: Colorectal liver metastases
ESCC: Esophageal squamous cell carcinoma
FPR: Fibrinogen to prealbumin ratio
HCC: Hepatocellular carcinoma
M: Multivariate
NR: Not reported
NSCLC: Non-small cell lung cancer
PFS: Progression-free survival
ROC: Receiver operating characteristic
U: Univariate
