# Supplementary material for: Albumin-fibrinogen ratio and fibrinogen-prealbumin ratio as promising prognostic markers for cancers: an updated meta-analysis
Source: World J Surg Oncol. 2020 Jan 13;18:9. doi: 10.1186/s12957-020-1786-2 (PMC6958612; doi:10.1186/s12957-020-1786-2)
Supplement: Supplementary file 1 — Additional file 1. Forest plots of the relationship between AFR and OS via multivariate analyses, before deleting the subgroup with paradoxical results (Li et al. 2019, subgroup 1). [file 12957_2020_1786_MOESM1_ESM.docx]

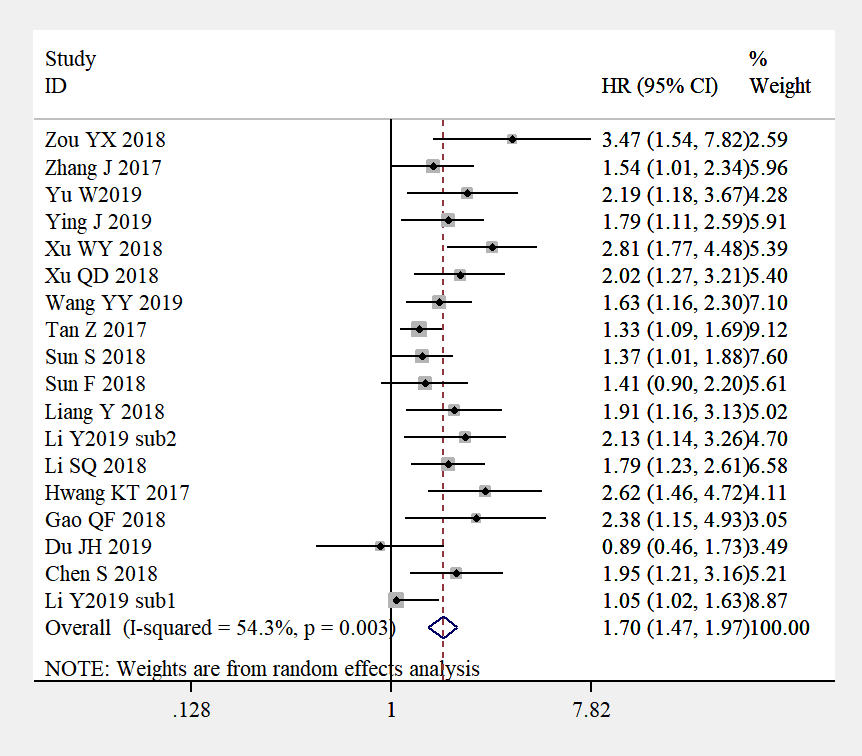


**Additional File 1.** Forest plots of the relationship between AFR and OS *via* multivariate analyses, before deleting the subgroup with paradoxical results (Li et al. 2019, subgroup 1).
