# Supplementary material for: Albumin-fibrinogen ratio and fibrinogen-prealbumin ratio as promising prognostic markers for cancers: an updated meta-analysis
Source: World J Surg Oncol. 2020 Jan 13;18:9. doi: 10.1186/s12957-020-1786-2 (PMC6958612; doi:10.1186/s12957-020-1786-2)
Supplement: Supplementary file 2 — Additional file 2. Sensitivity analysis of the correlation of AFR with OS via univariate analyses (A) and multivariate analyses (B); with DFS from univariate analyses (C) and multivariate analyses (D); and with PFS from univariate analyses results (E) and multivariate analyses (F). [file 12957_2020_1786_MOESM2_ESM.docx]

**A
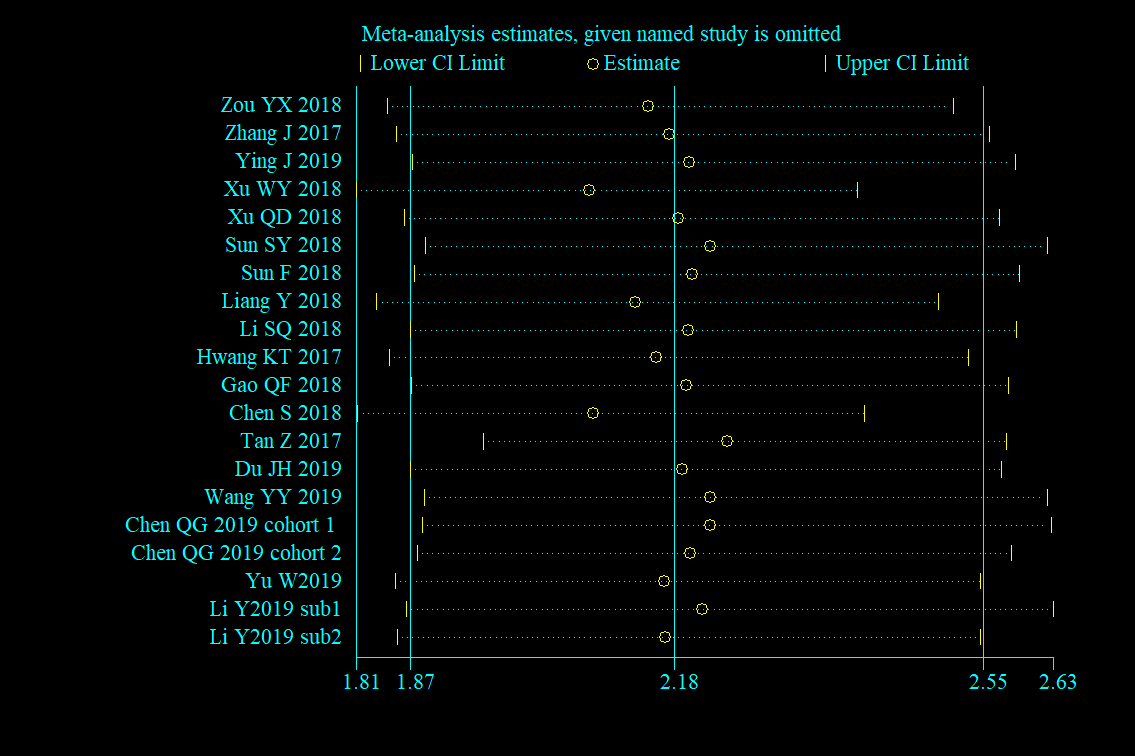
**

**B**
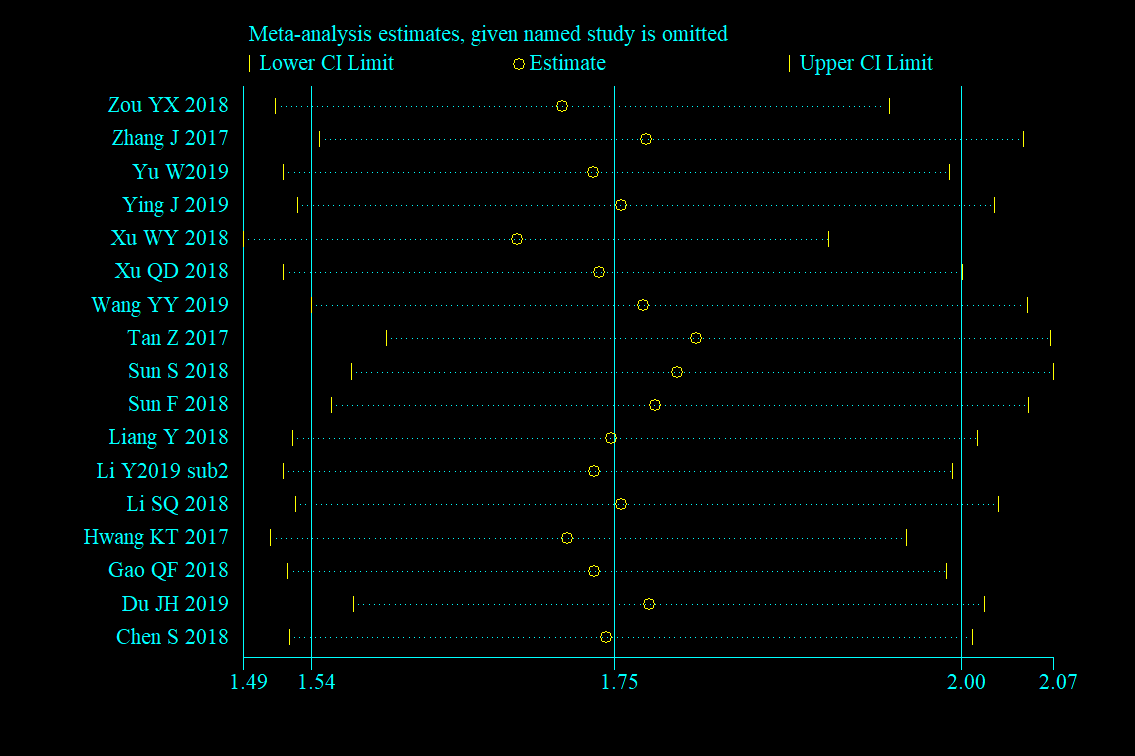


**C**
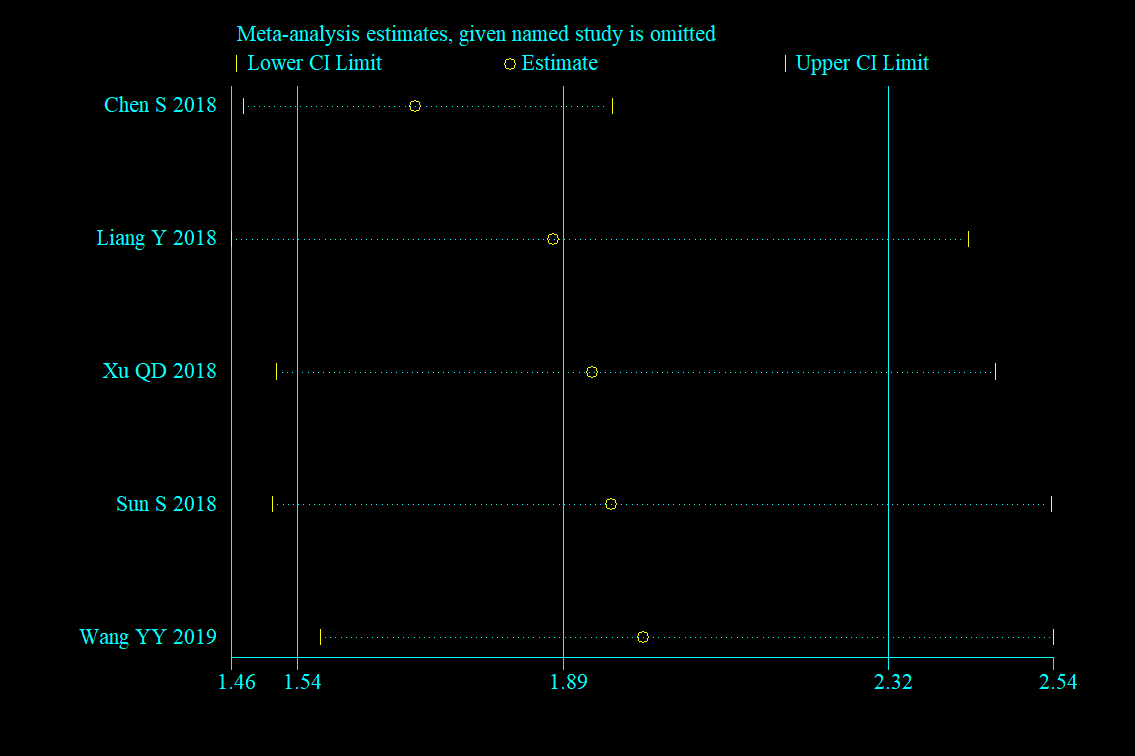


**D**
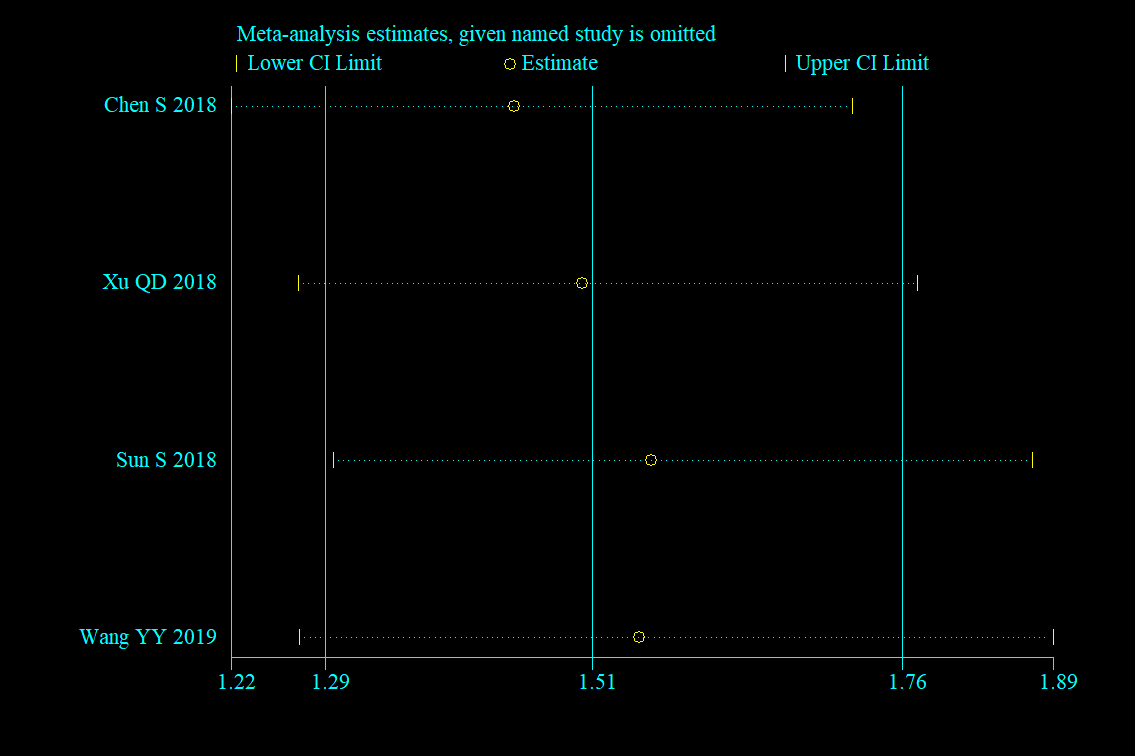


**E**
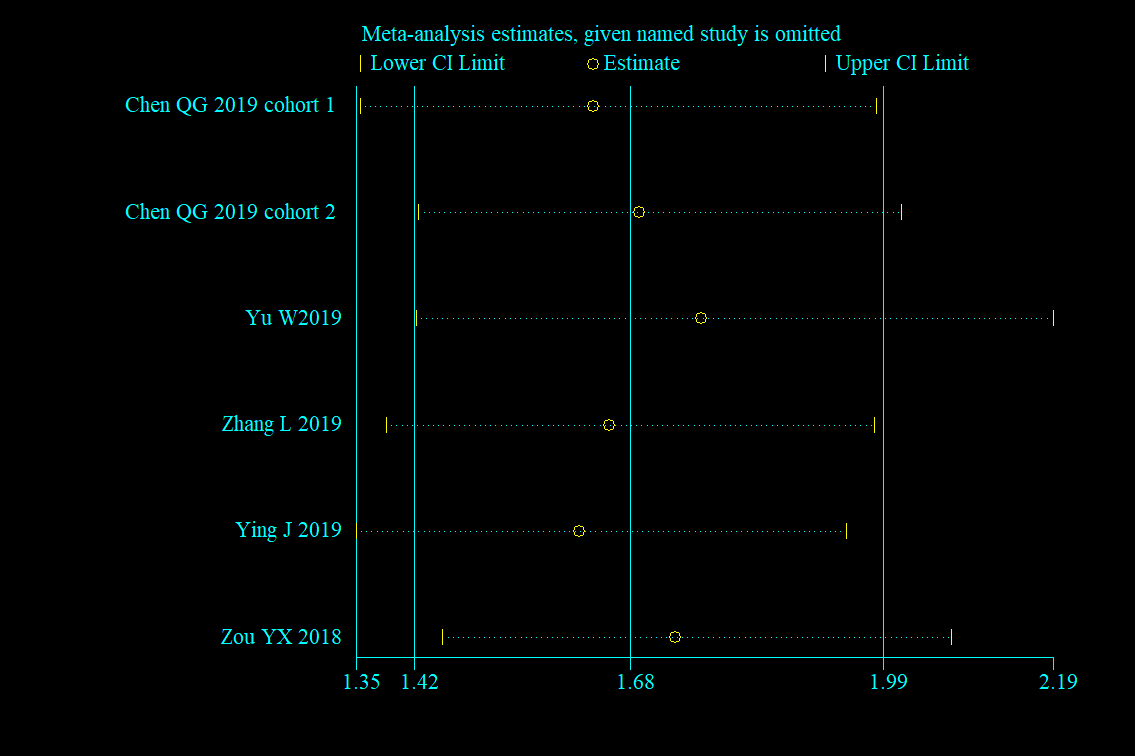


**F**
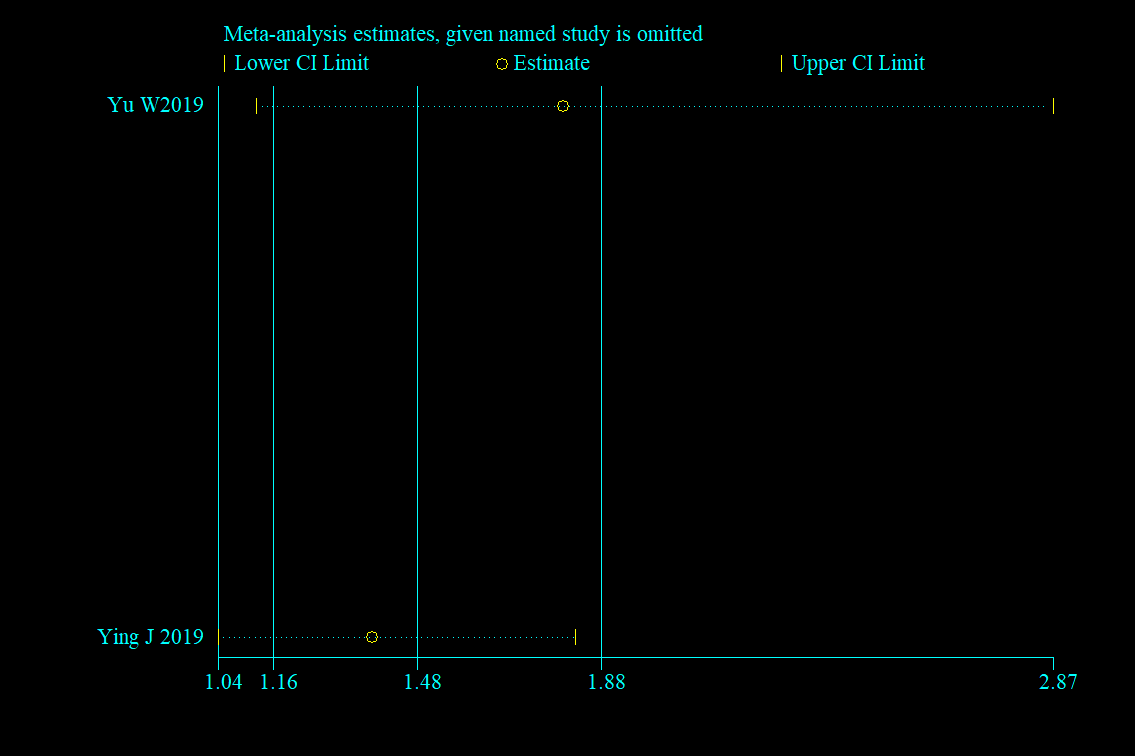


**Additional File 2.** Sensitivity analysis of the correlation of AFR with OS *via* univariate analyses (**A**) and multivariate analyses (**B**); with DFS from univariate analyses (**C**) and multivariate analyses (**D**); and with PFS from univariate analyses results (**E**) and multivariate analyses (**F**).
