# Supplementary material for: Albumin-fibrinogen ratio and fibrinogen-prealbumin ratio as promising prognostic markers for cancers: an updated meta-analysis
Source: World J Surg Oncol. 2020 Jan 13;18:9. doi: 10.1186/s12957-020-1786-2 (PMC6958612; doi:10.1186/s12957-020-1786-2)
Supplement: Supplementary file 3 — Additional file 3. Sensitivity analysis of the correlation of FPR with OS via univariate analyses (A) and multivariate analyses (B). [file 12957_2020_1786_MOESM3_ESM.docx]

**A**
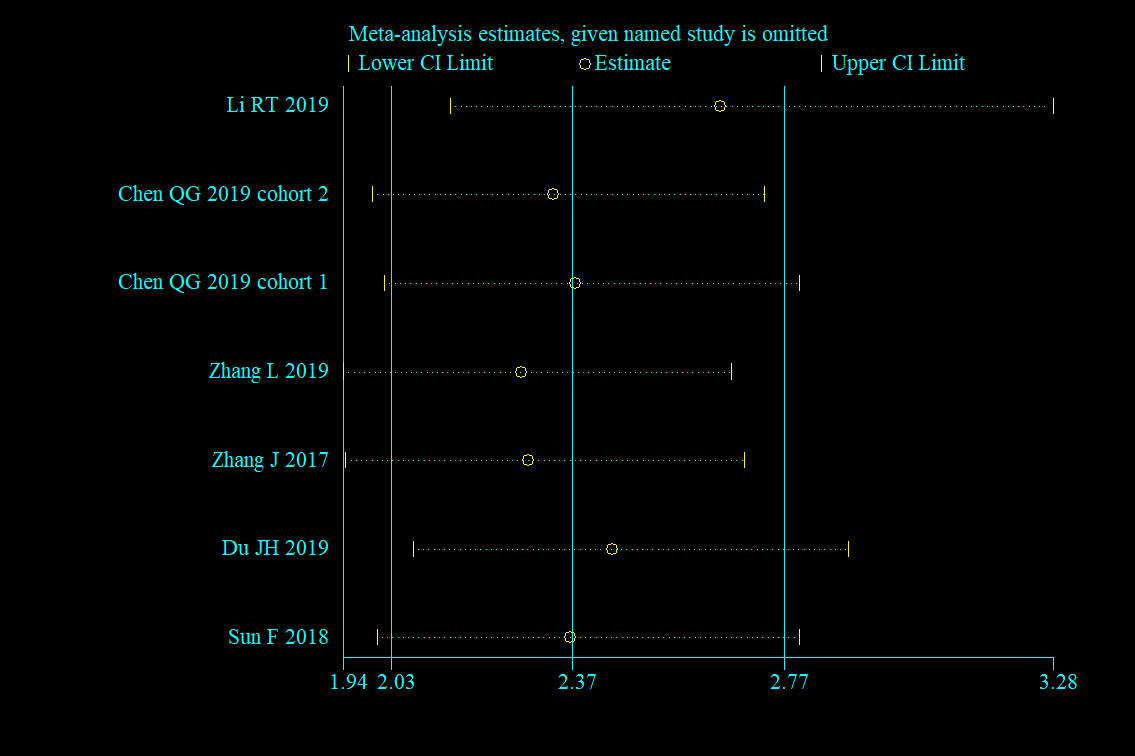


**B**
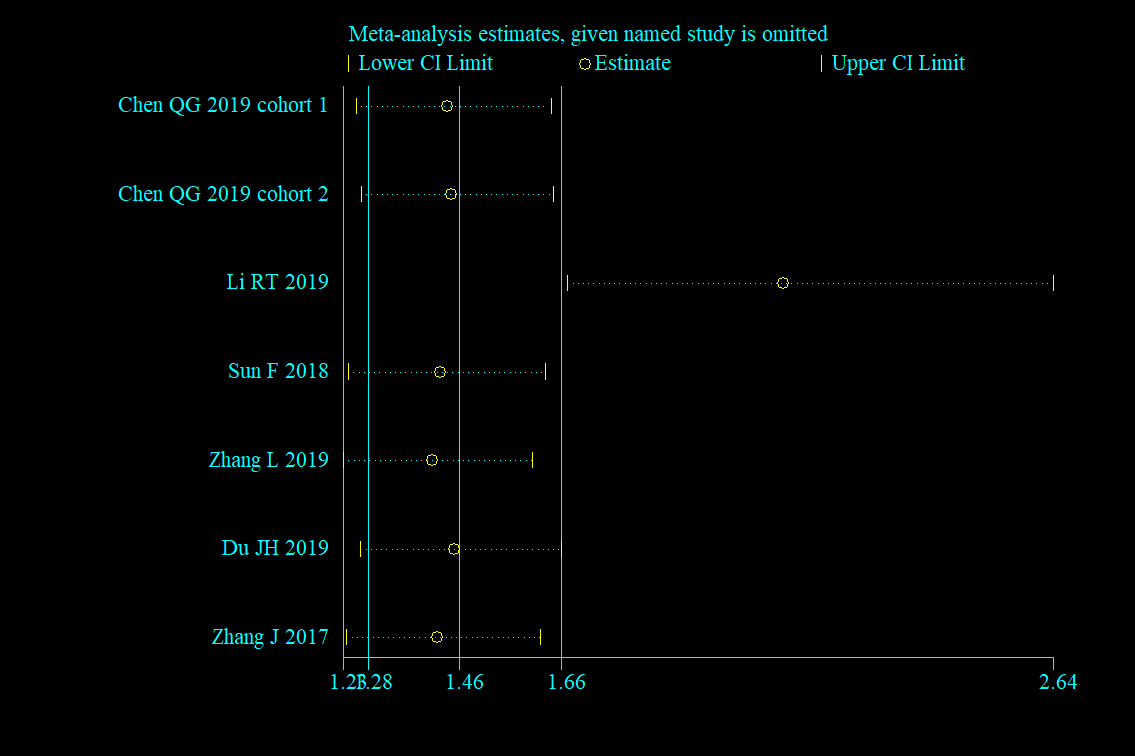


**Additional file 3.** Sensitivity analysis of the correlation of FPR with OS *via* univariate analyses (**A**) and multivariate analyses (**B**).
